# Morbidity and Mortality Due to *Bordetella pertussis*: A Significant Pathogen in West Africa?

**DOI:** 10.1093/cid/ciw560

**Published:** 2016-11-02

**Authors:** Beate Kampmann, Grant Mackenzie

**Affiliations:** 1 Medical Research Council Unit The Gambia, Fajara, The Gambia, West Africa, Banjul; 2 Centre for International Child Health, Imperial College London; 3 London School of Hygiene and Tropical Medicine, Faculty of Infectious and Tropical Diseases, Keppel Street, United Kingdom; 4 Murdoch Childrens Research Institute, Melbourne, Australia

**Keywords:** pertussis, West Africa, surveillance, case finding

## Abstract

In the absence of specific surveillance platforms for pertussis and availability of suitable diagnostics at the hospital level, reliable data that describe morbidity and mortality from pertussis are difficult to obtain in any setting, as is the case in West Africa. Here, we summarize the available evidence of the burden of pertussis in the region, given historical data, and describe recent and ongoing epidemiological studies that offer opportunities for additional data collection. The available seroepidemiological data provide evidence of ongoing circulation of *Bordetella pertussis* in the region. Due to the lack of systematic and targeted surveillance with laboratory confirmation of *B. pertussis* infection, we cannot definitively conclude that pertussis disease is well controlled in West Africa. However, based on observations by clinicians and ongoing demographic surveillance systems that capture morbidity and mortality data in general terms, currently there is no evidence that pertussis causes a significant burden of disease in young children in West Africa.

Despite the reported outbreaks of pertussis in high-income settings over recent years, relatively little data are available from West Africa to substantiate the World Health Organization's estimated figures for morbidity and mortality in low- and middle-income countries (LMICs) [1]. In West Africa, the current disease burden cannot be quantified accurately as no reliable and consistent surveillance systems for pertussis are in place.

Nevertheless, epidemiological data were collected in Senegal in the 1990s and, more recently, some epidemiological, seroepidemiological, and hospital-based data have been reported from Senegal, the Gambia, and Niger. We subsequently refer to these, albeit limited, settings as West Africa.

## HISTORICAL DATA FROM SENEGAL

**Figure 1.**
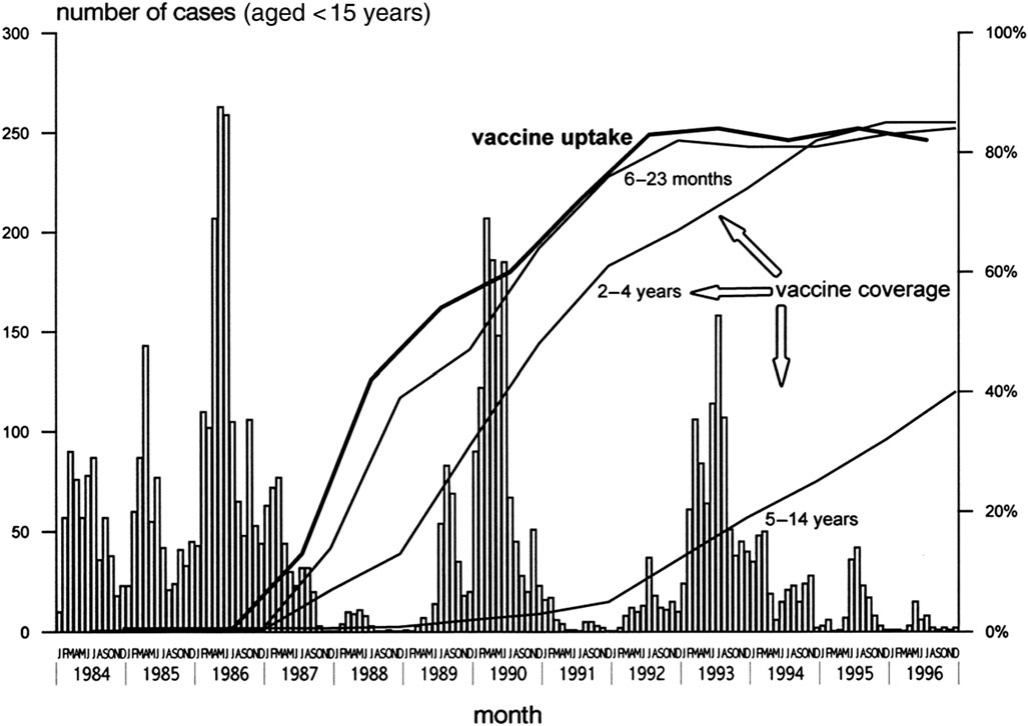
Pertussis cases per month, vaccine uptake and age-specific vaccine coverage per year, Niakhar, Senegal, 1984–1996. Figure reproduced with permission from Preziosi et al [2].

An active surveillance program for pertussis was implemented in Senegal's Niakhar community in the 1980s to mid-1990s, with the aim of measuring the changes in pertussis incidence before and after introduction of the whole cell pertussis vaccine into that community [2]. More than 15 000 children aged <15 years were actively followed over a 13-year period. The results showed a crude pertussis incidence before vaccination of 183 per 1000 child-years, with a 2.8% case-fatality rate (Table 1). After introduction of the vaccination program, overall incidence dropped by 27% after 3 years and by 46% after 6 years (Figure 1). The data clearly indicate the high efficacy of the whole cell vaccine on pertussis cases in the observed communities.

**Table 1.** Pertussis Case Distribution and Incidence per Age and Sex, During Epidemic Years, Niakhar, Senegal, 1984–1996

Age	First Outbreak (1986)				Second Outbreak (1990)				Third Outbreak (1993)			
	Cases		No. of PYR	Incidence per 1000 PYR	Cases		No. of PYR	Incidence per 1000 PYR	Cases		No. of PYR	Incidence per 1000 PYR
	No.	%			No.	%			No.	%		
0–5 mo	97	7	582	166.6	68	6	557	122.1	38	4	575	66.1
6–23 mo	246	18	1443	170.5	144	12	1700	84.7	58	7	1598	36.3
2–4 y	492	35	2530	194.5	348	30	2850	122.1	241	27	2969	81.2
5–14 y	570	40	6481	88	612	52	7422	82.5	555	62	7811	71.1
Total (0–14 y)	1405	100	11 036	127.3	1172	100	12 529	93.5	892	100	12 953	68.9
Girls	721		5395	133.6	580		6093	95.2	462		6276	73.6
Boys	684		5641	121.3	592		6436	92.0	430		6677	64.4
Relative risk (girls/boys) (95% confidence interval)	1.1 (1.0, 1.2)				1.0 (.9, 1.2)				1.1 (1.0, 1.3)			

Reproduced with permission from Preziosi et al [2].

Abbreviation: PYR, person-years at risk.

This surveillance activity was discontinued after 1996 and no further follow-up information is available. However, recent Senegalese population-based data reveal no cases of pertussis disease in children aged <10 years (Dr A. Diallo, personal communication).

## HOSPITAL DATA FROM NIGER

A recently published, hospital-based study from Niger reported results from the analysis of nasopharyngeal aspirates (NPAs) from 324 children hospitalized with respiratory symptoms at the National Hospital of Niamey [3]. The NPAs were tested by both culture and polymerase chain reaction (PCR). Of the 305 samples included in the analysis, 34 (11.2%) were found to be positive for pertussis on PCR; however, only a single sample was also positive on bacterial culture. The authors concluded that there was an overall pertussis prevalence of 8.2 cases/1000 children in this highly selective population of hospitalized children.

## SEROEPIDEMIOLOGY IN THE GAMBIA AND SENEGAL

Currently no program of pertussis-specific surveillance exists in West Africa. Recently we conducted a large cross-sectional seroepidemiological study in the Gambia [4]. We accessed stored samples from more than 1000 individuals aged between 2 and 90 years and measured immunoglobulin (Ig)G-pertussis toxin (PTx) using luminex xMAP technology [5]. IgG-PTx concentrations have been widely used to assess the prevalence of *B. pertussis* infections. Previous studies show that concentrations of anti-PTx antibodies ≥62.5 and ≥125 EU/mL were evidence of a previous infection in the past 12 and 6 months, respectively [6].

IgG-PTx geometric mean concentrations (GMCs) and their 95% confidence intervals were calculated. The proportion seropositive (>20 EU/mL or ≥62.5 EU/mL) and GMCs were compared based on age, sex, ethnic group, vaccination status, birth order, and number of siblings per household using logistic and linear regression.

**Figure 2.**
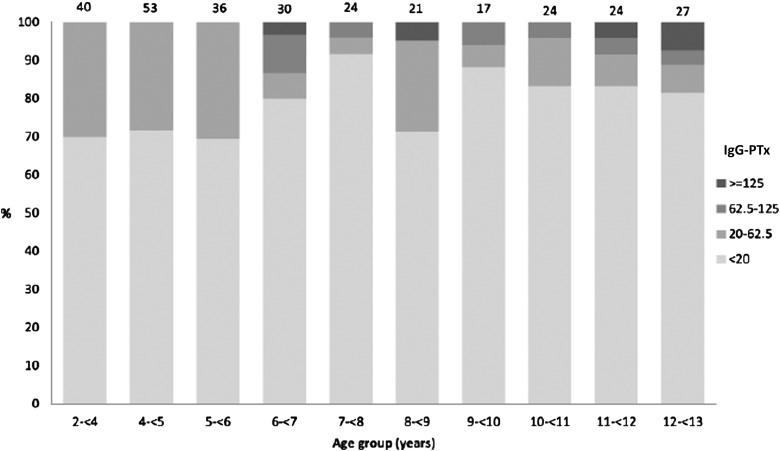
Concentrations of pertussis toxin (PTx) immunoglobulin G by age group for those with vaccination records (born after 1 January 1996), 2008 serosurvey in Keneba and Manduar, West Kiang region, the Gambia. Numbers on top of bars are total number sampled by age group. Reproduced with permission from Scott et al [4].

**Figure 3.**
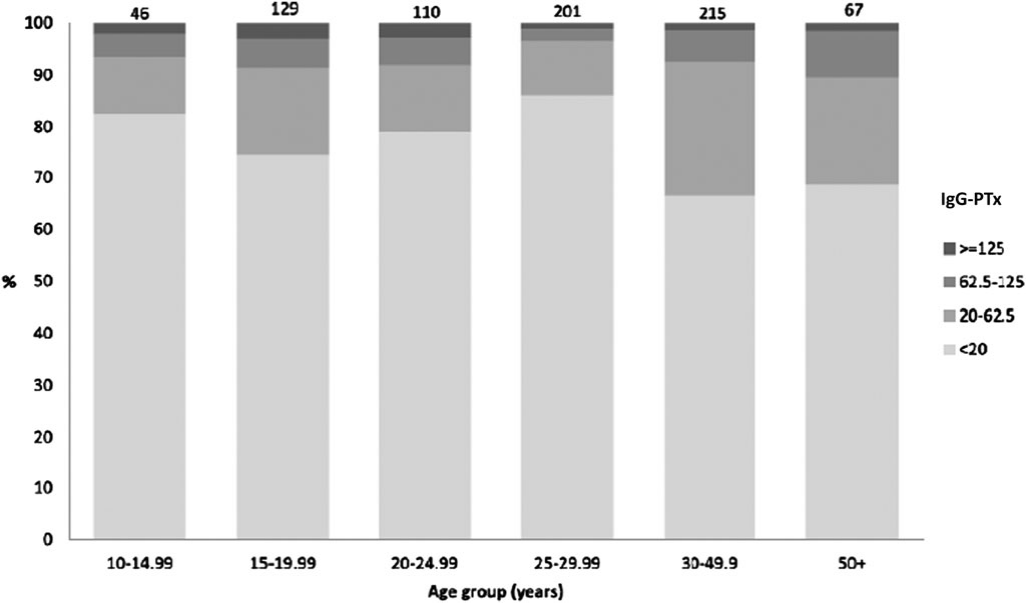
Concentrations of pertussis toxin (PTx) immunoglobulin G by age group for those without vaccination records (born after 1 January 1996), 2008 serosurvey in Keneba and Manduar, West Kiang region, the Gambia. Numbers on top of bars are total number sampled by age group. Figure reproduced with permission from Scott et al [4].

The Expanded Programme of Immunization (EPI) in the Gambia, as in most LMICs, includes a whole cell pertussis antigen as part of the pentavalent vaccine (hepatitis B, *Haemophilus influenzae* type b, diphtheria–pertussis–tetanus [DPT]). Coverage of 3 doses of pentavalent vaccine by 12 months of age was 94% in 2014. In this collection of samples, which was collected and stored between 2005 and 2012, 77% of the cohort had received at least 3 doses of DPT prior to sampling. Our results showed that 6% of individuals had antibody concentrations ≥62.5 EU/mL, which is indicative of a recent pertussis infection (Figures 2 and 3).

Unfortunately, no stored samples were available from children aged <2 years, hence we cannot comment on evidence of responses to either vaccination or environmental exposure in infants and children aged <2 years. We also analyzed a small set of 30 cord blood samples for PTx antibody titers and saw no evidence of recent exposure of pregnant women, as all titers were below 20 EU/mL (B. Kampmann, unpublished data). These data are consistent with findings in Thailand [7] and Sweden [8], which also did not show evidence of recent infection in pregnant women based on PTx antibody levels. To extend the available dataset relating to women of childbearing age as a possible source of infection in infants, a larger evaluation of PTx in stored cord blood samples could be undertaken relatively easily as a proxy. However, our age-related longitudinal data presented above do not indicate that there is a particular peak in antibody titers that is suggestive of recent infection in this age group, possibly because of ongoing protection via the whole cell vaccine received in childhood. This hypothesis could be tested in a comparable cohort of women who were immunized with acellular pertussis vaccine in childhood. However, this is not the case in West Africa, and differences in epidemiological background might play a role in the interpretation of such comparative studies.

A relatively recent seroepidemiological study included 402 children aged between 1 and 9 years from 5 villages in northern Senegal for longitudinal measurements of the IgG response to PTx over a 15-month period, during which 5 blood samples were collected from each child at regular intervals [9]. More than 85% of the cohort had documented evidence of having received at least 1 dose of diptheria, tetanus and whole cell pertussis; however, vaccination coverage differed between the sites, correlating with the antibody titers measured. A total of 33.6% of the global cohort presented anti-PTx IgG concentrations higher than 30 IU/mL and 16.2% higher than 80 IU/mL. In this study, circulation of *B. pertussis* was clearly shown by serological measurement, with great variation in serological responses to PTx antigens according to age and village of residence. However, the authors did not report cases of pertussis disease among this cohort.

## OPPORTUNITIES TO GATHER DATA FROM EXISTING PNEUMONIA SURVEILLANCE PLATFORMS AND DEMOGRAPHIC HEALTH SURVEILLANCE SYSTEMS

In the absence of a dedicated pertussis surveillance platform, data from other field activities might be informative. The best data on vaccination and serious infections in infancy in our region are from the Basse Health & Demographic Surveillance System (BHDSS) in the rural east section of the Gambia [10]. The surveillance area encompasses a geographic area of 1111 km^2^, with a total population of 180 168 at the end of 2015 and 6475 infants aged 0–11 months. The EPI in the Gambia includes a whole cell pertussis antigen as part of the pentavalent vaccine (hepatitis B, *H. influenzae* type b, DPT). In the BHDSS, coverage of 3 doses of pentavalent vaccine by age 12 months was 94% in 2014. Verbal autopsies (a research method that helps to determine probable causes of death in cases without medical records or formal medical examination) are performed for all deaths in the BHDSS, which are interpreted using the Inter-VA algorithm (interva.net). Diagnosis of pertussis using the Inter-VA algorithm has not been validated.

Population-based surveillance for suspected meningitis, sepsis, and pneumonia in individuals aged ≥2 months has been underway since 2008 [11]. The sensitive inclusion criteria would include children with clinical disease due to pertussis infection. Standardized identification and investigation of patients includes blood culture, chest radiograph, lumbar puncture, and other sterile site samples. Nasopharyngeal swabs (NPS) have been collected from all patients who met surveillance criteria since 2015. The meningitis, sepsis, and pneumonia surveillance system was expanded in 2012 to investigate by blood culture all children aged <5 years who were admitted to health facilities in the BHDSS. The BHDSS surveillance for invasive bacterial disease in pediatric admissions and meningitis, sepsis, and pneumonia in patients aged ≥2 months is ongoing in 2016.

Child mortality in the BHDSS is substantial, with 60–70 per 1000 live births; there were 367 deaths in infancy in 2014/2015 and 137 deaths occurred in health facilities. However, the surveillance system described above was not designed to detect cases of infection due to *B. pertussis*. Microbiological investigations are focused on common invasive bacterial pathogens, with an aim to determine the impact of the introduction of pneumococcal conjugate vaccine. NPSs are primarily investigated for pneumococcal carriage. Laboratory investigations currently available on site do not include specific PCR for the detection of *B. pertussis* or *Bordetella parapertussis*.

Clinical findings are recorded for all patients who meet surveillance criteria. Clinical diagnoses that may overlap with pertussis infection in children aged 0–23 months include a clinician's diagnosis of pertussis, whooping cough, croup, and apnea in infants aged <2 months. The surveillance system enrolled 13 192 children aged 0–23 months from 12 May 2008 to 31 October 2015. The reported diagnoses included pertussis in 4 cases, croup in 6, and apnea in 3. Thus, there is little clinical evidence of significant pertussis transmission in this population.

## DATA FROM THE GAMBIA SITE OF THE PNEUMONIA ETIOLOGY RESEARCH FOR CHILD HEALTH PROJECT

Despite the BHDSS surveillance system's lack of specific diagnosis for pertussis, some laboratory data are available from that location. The Pneumonia Etiology Research for Child Health (PERCH) project was located in the BHDSS in 2012–2013. Approximately 650 children aged 1–59 months with acute cough and either chest wall indrawing or clinical danger signs were investigated. This case definition may overlap with that of pertussis infection. Children were investigated using nasopharyngeal and oropharyngeal swabs, which were analyzed using a multiplex real-time PCR assay that included *B. pertussis*. (For full details of the PERCH study, see article by Kate O′Brian in this supplement.) In the 2 years of enrollment for PERCH in the Gambia, pertussis was detected in 3 cases using this assay. These data indicate that pertussis is not a common cause of clinical pneumonia in our setting.

## CURRENT DATA FROM HOSPITALIZED CHILDREN IN THE GAMBIA AND SENEGAL

A recently published audit of morbidity and mortality in neonates in the main teaching hospital in the Gambia reported morbidity and mortality of nearly 5000 neonates admitted during a 5-year period to assess outcome [12].

Of the 4944 admissions between 1 January 2010 and 31 December 2013, 1734 infants (35%) died, with 57% of all deaths occurring within the first 48 hours of admission. Possible severe bacterial infection accounted for 44% (2166/4944) of admissions, prematurity/low birthweight for 27% (1340/4944), and intrapartum-related conditions for 20%. Pertussis was not recorded as a cause of death in any of the cases; however, investigations to ascertain causative organisms in any of the neonates were minimal. Although this represents a comprehensive dataset, the question of whether pertussis contributed to morbidity or mortality in these infants in the neonatal period cannot be answered conclusively.

Considering evidence of pertussis disease beyond the borders of the Gambia, we have worked with colleagues at Centre Hospitalier Régional Kaolack in central Senegal. After the capital city Dakar, Kaolack is the second largest city in Senegal. The regional hospital is a referral center for central Senegal, offering a range of specialty services. In 2014, 2932 children were admitted to pediatric services and 3873 were admitted in 2015. The clinical severity of admitted cases is high, with 1298 (19%) deaths recorded among children admitted in 2014 and 2015. A significant proportion of pediatric admissions are in the neonatal period (24%, 1602/6805). Of all pediatric deaths, 21% (278/1298) occurred in the neonatal period with prematurity (n = 127), asphyxia (n = 79), and infection (n = 72) the predominant causes of mortality. Of all pediatric admissions in Kaolack, 14% are associated with malnutrition, 9% are due to acute respiratory infections, 8% malaria, 7% diarrhea, and 24% of neonatal admissions are associated with infection. Pertussis is an uncommon diagnosis, with little evidence that it plays a significant role in the burden of illness in childhood (Dr B. Mbodj, personal communication).

## SO, IS PERTUSSIS A SIGNIFICANT PATHOGEN IN WEST AFRICA?

In order to answer the question of whether pertussis is a significant pathogen in the Gambia and Senegal, one must consider possible reasons for cases being missed and the likelihood of these scenarios. The primary reason that our data may miss cases of pertussis is that many children with acute illness do not present to health facilities. A secondary reason is clinical misdiagnosis of cases of pertussis as an alternative respiratory illness such as bronchopneumonia, bronchiolitis, or other lower respiratory tract infections, which is a common problem. In a recent publication from South Africa [13], up to 90% of pertussis cases were assigned an alternative diagnosis. Despite reasonable immunization coverage by age 12 months in our setting, delayed immunization is common, and cases of pertussis may follow the usual epidemiology, with cases occurring before vaccine-induced protection is achieved.

However, the clinical experiences in Basse and Kaolack suggest that pertussis is quite uncommon in infants presenting to health facilities. The clinicians in both settings are experienced pediatricians who would be expected to detect the classic features of whooping cough. Misdiagnosis seems unlikely as atypical cases of pertussis in infancy are unlikely in the absence of significant numbers of typical cases. More likely than misdiagnosis, it seems that cases of pertussis may be missed among infants who do not present to health facilities. However, in our experience, childhood deaths at home often follow an initial presentation to health facilities. In the BHDSS, 44% of neonatal deaths occur at home without a specific diagnosis. Thus, in Basse and Kaolack, it is possible that the paucity of pertussis cases may relate to children not presenting to health facilities.

Despite our clinical experience, due to the lack of systematic and targeted surveillance with laboratory confirmation of *B. pertussis* infection, we cannot definitively conclude that pertussis disease is well controlled in West Africa.

## SUGGESTED INTERVENTIONS TO CONCLUDE ON THE BURDEN OF PERTUSSIS IN WEST AFRICA

To provide certain resolution to the question of whether there is significant transmission of pertussis in our setting, 2 relatively simple surveillance techniques could be added to our current procedures. First, for a period of 2 years, an NPS could be taken from every child aged <12 months admitted to medical facilities in Basse and Kaolack with cough as a presenting symptom. Induced sputum appears to have a slightly higher yield to detect the pathogen, either on its own or in combination with NPS [13]. However, in the context of larger surveillance studies, this is less practical. Second, active surveillance could be conducted in 2 or 3 easily accessible urban areas with variable vaccination coverage or, more specifically, varied delay in the receipt of EPI vaccines. Suitable urban areas include Basse (medium/high vaccine coverage), Kaolack (medium vaccine coverage), and 1 other more isolated setting with more modest vaccine coverage, as available from our DSS data. Samples would need to be processed using a sensitive PCR assay, optimized for the detection of *B. pertussis* or *B. parapertussis*, since culture methods are known to lack sensitivity [14]. If pertussis transmission was found to be significant and these surveillance measures were taken, one would expect to detect substantial numbers of laboratory-confirmed cases. If these relatively intense surveillance measures failed to detect significant numbers of pertussis cases, one would conclude that good disease control has been achieved.
